# Targeting influenza A virus by splicing inhibitor herboxidiene reveals the importance of subtype-specific signatures around splice sites

**DOI:** 10.1186/s12929-023-00897-4

**Published:** 2023-02-03

**Authors:** Yi-Ju Han, Kuo-Ming Lee, Guan-Hong Wu, Yu-Nong Gong, Avijit Dutta, Shin-Ru Shih

**Affiliations:** 1 Graduate Institute of Biomedical Science, College of Medicine, Chang Gung University, Taoyuan, Taiwan; 2 Research Center of Emerging Virus Infection, Division of Biotechnology, College of Medicine, Chang Gung University, Taoyuan, Taiwan; 3 International Master Degree Program for Molecular Medicine in Emerging Viral Infections, College of Medicine, Chang Gung University, Taoyuan, Taiwan; 4 Division of Infectious Diseases, Department of Pediatrics, Linkou Chang Gung Memorial Hospital, Taoyuan, Taiwan; 5 Department of Laboratory Science, Linkou Chang Gung Memorial Hospital, Taoyuan, Taiwan; 6 Division of Infectious Diseases, Department of Medicine, Linkou Chang Gung Memorial Hospital, Taoyuan, Taiwan; 7 Department of Medical Biotechnology and Laboratory Science, College of Medicine, Chang Gung University, Taoyuan, Taiwan; 8 Research Center for Chinese Herbal Medicine, Chang Gung University of Science and Technology, Taoyuan, Taiwan; 9 Research Center for Food and Cosmetic Safety, Chang Gung University of Science and Technology, Taoyuan, Taiwan; 10 Graduate Institute of Health Industry Technology, College of Human Ecology, Chang Gung University of Science and Technology, Taoyuan, Taiwan

**Keywords:** Influenza A virus, Pathogenicity, Splicing, Herboxidiene, Subtype-specific signature, M2 protein

## Abstract

**Background:**

The association between *M* segment splicing and pathogenicity remains ambiguous in human influenza A viruses. In this study, we aimed to investigate *M* splicing in various human influenza A viruses and characterize its physiological roles by applying the splicing inhibitor, herboxidiene.

**Methods:**

We examined the *M* splicing of human H1N1 and H3N2 viruses by comparing three H1N1 and H3N2 strains, respectively, through reverse transcriptase-polymerase chain reaction (RT-PCR) analyses. We randomly selected *M* sequences of human H1N1, H2N2, and H3N2 viruses isolated from 1933 to 2020 and examined their phylogenetic relationships. Next, we determined the effects of single nucleotide variations on *M* splicing by generating mutant viruses harboring the 55C/T variant through reverse genetics. To confirm the importance of *M2* splicing in the replication of H1N1 and H3N2, we treated infected cells with splicing inhibitor herboxidiene and analyzed the viral growth using plaque assay. To explore the physiological role of the various levels of M2 protein in pathogenicity, we challenged C57BL/6 mice with the H1N1 WSN wild-type strain, mutant H1N1 (55T), and chimeric viruses including H1N1 + H3wt and H1N1 + H3mut. One-tailed paired *t*-test was used for virus titer calculation and multiple comparisons between groups were performed using two-way analysis of variance.

**Results:**

*M* sequence splice site analysis revealed an evolutionarily conserved single nucleotide variant C55T in H3N2, which impaired *M2* expression and was accompanied by collinear *M1* and *mRNA3* production. Aberrant *M2* splicing resulted from splice-site selection rather than a general defect in the splicing process. The C55T substitution significantly reduced both M2 mRNA and protein levels regardless of the virus subtype. Consequently, herboxidiene treatment dramatically decreased both the H1N1 and H3N2 virus titers. However, a lower M2 expression only attenuated H1N1 virus replication and in vivo pathogenicity. This attenuated phenotype was restored by *M* replacement of H3N2 *M* in a chimeric H1N1 virus, despite low M2 levels.

**Conclusions:**

The discrepancy in M2-dependence emphasizes the importance of M2 in human influenza A virus pathogenicity, which leads to subtype-specific evolution. Our findings provide insights into virus adaptation processes in humans and highlights splicing regulation as a potential antiviral target.

## Background

Influenza A viruses (IAVs) of the Orthomyxoviridae family infect various species, including mammals and birds. Being associated with seasonal influenza (flu) epidemics, IAVs have caused several pandemics worldwide, including the 1918 Spanish flu, which resulted in 50 million deaths [1]. IAV is an enveloped virus carrying a segmented genome that comprises eight negative-sense and single-stranded RNA segments. Each segment is wrapped by a nucleoprotein (NP) into a viral ribonucleoprotein (RNP) complex along with a viral RNA polymerase complex comprising polymerase basic 1 (PB1) polymerase, cap‐binding PB2 subunit, and polymerase acidic (PA) endonuclease. The surface glycoproteins hemagglutinin (HA) and neuraminidase (NA) involved in receptor binding and virus release are used to classify IAVs. To date, 18 subtypes of HA (H1–18) and 11 of NA (N1–11) have been identified, but only H1N1, H2N2, and H3N2 are routinely identified in humans [2, 3].

Multiple proteins are generated by the splicing of transcripts of segments 1, 7, and 8 (also known as *PB2*, *M*, and *NS* segments, respectively) [4–7]. Splicing of the *NS* segment, which encodes the host shutoff protein non-structural protein 1 (NS1), generates the nuclear export protein (NEP/NS2) that is involved in viral RNP export from the host nucleus [8]. Alternative splicing of the *M* segment produces collinear *M1*, spliced *M2*, *mRNA3*, and *M4*. M1 is a structural matrix protein that stabilizes viral particles, whereas the ion channel protein M2 is embedded in the viral envelope and regulates the uncoating of vRNP [9]. M2 can be replaced by the M42 protein encoded by *M4*; however, *M4* can only be detected in some H1N1 virus strains, such as A/WSN/33 [10, 11]. Splicing of PB2 generates PB2-S1, which was only identified in pre-2009 human H1N1, and may inhibit the retinoic acid-inducible gene I signaling pathway [4].

Both genetic drift (i.e., mutation) and genetic shifts (i.e., reassortment) contribute to IAV evolution and might allow cross-species infection in humans, which result in pandemics [12]. Four major flu pandemics—the 1918 H1N1, 1957 H2N2, 1968 H3N2, and the swine-origin 2009 pandemic H1N1 (pdmH1N1)—were associated with the switch of the prevalent strain, that is, from H2N2 to H3N2 and from pre-2009 H1N1 to pdmH1N1 [13–16]. Therefore, combatting IAVs is a never-ending challenge, and seasonal flu continues to threaten humans. Hundreds of thousands of deaths are caused by seasonal flu every year, and the estimated number of annual deaths worldwide in 1999–2015 ranged from 291,243 to 645,832 [17]. H1N1 and H3N2 are the main etiologies of seasonal flu. In Taiwan, the reproduction rate of seasonal H3N2 and H1N1 viruses is 1.41 and 1.19, respectively, and the H3N2 virus had a higher transmissibility than the H1N1 virus [18]. Before the 2009 H1N1 pandemic, 71% of seasonal flu‐attributable deaths in the United States were caused by H3N2 [19]. Although H3N2 might have a higher transmissibility and attack rate, no H3N2 pandemic has been reported since 1968. Besides, its specific pathogenesis remains unexplored.

The co-circulation of H1N1 and H3N2 raises another concern regarding reassortment. Laboratory experiments showed that reassortant virus progenies tend to be attenuated [20], and analyses of their genomes revealed a strong preference for PB2-PA combinations of the same genotype, whereas the H3N2 NA and H1N1 M segments are generally favored. Moreover, an authentic H3N2 variant carrying the pdmH1N1 M segment was identified in the United States [21]. The M segment preference is noteworthy because *M* splicing has been proposed to affect host range determinants and adaptation [22–24]. Various isoforms of the M segment are produced by utilizing the alternative 5′ splice site (5′ SS) [25]. Unlike *NS* splicing, in which a constant ratio of approximately 10% for spliced–collinear mRNA is observed throughout the infection [26], *M* splicing changes over time, and the initially dominant *M1* transcripts are gradually replaced by *M2* mRNA [27]. In addition to its involvement in the uncoating of vRNP, M2 participates in the inflammasome and autophagy pathways [28–30]. Therefore, the highly conserved M2 has been considered as a pivotal therapeutic target [31, 32].

Intriguingly, the splicing efficiency of *M* varies in a time-, species-, and strain-specific manner, and is coordinated by both host and viral proteins [33, 34]. Binding of the viral RNA polymerase complex to the 5′ terminus hinders the distal 5′ SS, resulting in the selection of the proximal *M2* 5′ SS [35]. NS1 functions similarly by spatially obstructing the distal 5′ SS and altering *mRNA3* splicing [36]. Conversely, selection of the weak proximal 5′ SS is mediated by host splicing factors, including serine/arginine-rich splicing factor 1 (SRSF1), influenza virus NS1-binding protein (NS1-BP), heterogeneous nuclear ribonucleoprotein K (hnRNP K), and transformer-2 protein homolog alpha (TRA2A) [22, 37, 38]. Hijacking of the splicing machinery can be achieved through the splicing regulator, dual specificity protein kinase CDC-like kinase 1 (CLK1), and small compounds inhibiting CLK-1 can impair IAV replication [39]. Regulatory elements in the *M* transcript have been identified and their recognition by species-specific host factors to alter *M* splicing of human and avian viruses can restrict cross-species infection [24]. However, whether *M* splicing in different human IAV subtypes differs and leads to different virulence levels remains unclear. Therefore, we specifically investigated the *M* splicing of various human IAVs and examined the effects of subtype-specific *cis-* and *trans*-regulators. We also characterized the physiological roles of *M* splicing by applying the splicing inhibitor, herboxidiene, and examining the in vivo pathogenic impact of M2.

## Methods

### Cell culture and transfection

Madin–Darby canine kidney (MDCK), chicken embryo fibroblast (DF-1), and human embryonic kidney 293 (HEK293) cells were obtained from the American Type Culture Collection and maintained in Dulbecco’s Modified Eagle Medium (DMEM) (Catalog number: 12100061; Thermo Fisher Scientific, Waltham, MA, USA) supplemented with 10% fetal bovine serum (FBS) (Part number: SH30396.03; Cytiva, Marlborough, MA, USA), 1% nonessential amino acid (Catalog number: 11140050; Thermo Fisher Scientific), and penicillin–streptomycin (Catalog number: 10378016; Thermo Fisher Scientific). Adenocarcinomic human alveolar basal epithelial cells (A549) were obtained from the American Type Culture Collection and maintained in minimal essential medium (MEM) (Catalog number: 11012044; Thermo Fisher Scientific) supplemented with 10% FBS, 1% nonessential amino acid, and penicillin–streptomycin. HEK293 cells were transfected with the indicated plasmids using Lipofectamine 2000 (Catalog number: 11668019; Thermo Fisher Scientific) according to the manufacturer’s instructions. At 48 h post-transfection, cell lysates and total RNA were collected.

### Viruses and reverse genetics plasmids

All viruses used in this study were generated using reverse genetics (RG) techniques. To generate the RG A/WSN/33 (H1N1) virus, 12 plasmids were used: PB2, PB1, PA, and NP cDNA were cloned into the pcDNA3 expression vector to synthesize the viral RNA (vRNA), and PB2, PB1, PA, HA, NP, NA, M, and NS cDNA were cloned into the pol1 vector system via BsmBI, as described by Fordor et al. [40]. The plasmids were kindly provided by George G. Brownlee (Lincoln College). The A/TW/2032/2017 (H3N2) and A/TW/3446/2002 viruses were clinical isolates obtained from Chang Gung Memorial Hospital and the RG plasmids were obtained as described by Hoffmann et al. [41]. The eight plasmids used to reconstitute the A/TW/3773/2015 (pH1N1) and A/Udorn/1972 RG viruses were gifted by Dr. Chung-Guei Huang (Chang Gung Memorial Hospital) and Dr. Rei-Lin Kuo (Chang Gung University), respectively.

For virus rescue, HEK293 (1 × 10^6^ cells) were co-transfected with twelve WSN or eight H3N2 plasmids using Lipofectamine 2000 Transfection Reagent. After 24 h, the medium was replaced with serum-free medium. The supernatant of the transfected cells was harvested at 3- and 5-days post-transfection and was then transferred to a 25-cm^2^ flask seeded with subconfluent MDCK cells to amplify any rescued viruses.

### Infection and growth curve analysis

HEK293 and DF-1 cells were infected with the RG viruses at a multiplicity of infection (MOI) of 1 in serum-free DMEM supplemented with trypsin (0.1 μg/mL) (Catalog number: 15050065; Thermo Fisher Scientific). To assess the growth curve of the viruses, A549 cells were infected with the RG viruses at a MOI of 0.001 or 1 in serum-free DMEM. After adsorption of viruses for 1 h at 4 °C, the cells were washed with phosphate-buffered saline (PBS) and maintained in serum-free DMEM supplemented with trypsin (0.2 μg/mL) at 37 °C for various time points. Then supernatants were harvested to determine the virus titer by plaque assay as described by Karakus et al. [42]. Briefly, 8 × 10^5^ MDCK cells were infected by serially diluted supernatants from 10^–1^ to 10^–6^. Absorption was performed at 37 °C for 1 h followed by extensive wash using PBS, and cells were overlaid by 0.3% agarose gel in serum-free DMEM supplemented with trypsin (1 μg/mL) and incubated at 37 °C for 48 to 60 h. Cells were then fixed using 4% formaldehyde and plaques were visualized by staining with 0.5% crystal violet.

### Reverse transcriptase-polymerase chain reaction (RT-PCR)

Total RNA was extracted from virus-infected or plasmid-transfected HEK293 cells using TOOLSmart RNA Extractor (#DPT-BD24; BIOTOOLS, Taiwan) and treated with RQ1 RNase-Free DNase (#M6101; Promega, Madison, WI, USA) at 37 °C for 30 min. Total RNA was then reverse-transcribed using an oligo(dT) primer and ReverTra Ace reverse transcriptase (#TRT-101, Toyobo, Osaka, Japan). The cDNA products were subjected to PCR analysis using specific primers to detect different M transcripts. The sequences for the four primers used to detect M isoforms are listed in Table 1. Quantitative PCR primers for M1 were M1-F, M1-R and primers for M2 were M2-F, 1R (891-912); the primers for mRNA3 and M4 were described by Chiang et al. [43]. The primers for ACTB are listed in Table 1. Relative mRNA was determined using ACTB as the normalization factor.

**Table 1:** Primers used in this study

M isoforms primers	
1F (1–11)	5′-GGGGGAGCAAAAGCAG-3′
2F (26–45)	5′-ATGAGTCTTCTAACCGAGGT-3′
3F (819–839)	5′-TTGCACTTGATATTGTGGATT-3′
1R (891–912)	5′-CTTCCGTAGAAGGCCCTCTTTT-3′
Quantitative PCR primers	
M1-F	5′-CCTCAAAGCCGAGATCGC-3′
M1-R	5′-GGGCACGGTGAGCGTGAA-3′
M2-F	5′-GAGGTCGAAACGCCTATCAGAA-3′
ACTB-F	5′-GCTCGTCGTCGACAACGGCTC-3′
ACTB-R	5′-CAAACATGATCCTGGGTCATCTTCTC-3′
The primers designed for mutagenesis	
H1N1 C55T-F	5′-CCGAGGTCGAAACGTATGTTCTCTCTATCGTCC-3′
H1N1 C55T-R	5′-GGACGATAGAGAGAACATACGTTTCGACCTCGG-3′
H3N2 T55C-F	5′-CCGAGGTCGAAACGTACGTTCTCTCTATCGTCC-3′
H3N2 T55C-R	5′-GGACGATAGAGAGAACGTACGTTTCGACCTCGG-3′
H1N1 G740A-F	5′-TCTTGAAAATTTGCAGACCTATCAGAAACGAATGGG-3′
H1N1 G740A-R	5′-CCCATTCGTTTCTGATAGGTCTGCAAATTTTCAAGA-3′
H3N2 A740G-F	5′-TCTTGAAAATTTGCAGGCCTATCAGAAACGAATGGG-3′
H3N2 A740G-R	5′-CCCATTCGTTTCTGATAGGCCTGCAAATTTTCAAGA-3′

### Site-directed mutagenesis

Primers for PCR-based site-directed mutagenesis was listed in Table 1.

### Western blotting

Cells were lysed using a lysis buffer (25 mM Tris-base, pH 7.6, 150 mM NaCl, 1% NP-40, 1% sodium deoxycholate, and 0.1% sodium dodecyl sulfate). The total protein extracts were then separated on 12% acrylamide gels, transferred to a polyvinylidene fluoride membrane, and detected using polyclonal antibodies against M1 (dilution factor: 1:1000), M2 (dilution factor: 1:2000), PA (dilution factor: 1:1000), and LC3 (dilution factor: 1:2000) (Catalog numbers: GTX127356, GTX125951, GTX118991, and GTX127375, respectively; GeneTex, Irvine, CA, USA) and monoclonal antibodies against actin (dilution factor: 1:4000) (Catalog numbers: MABT825; Millipore, Burlington, MA, USA).

### Herboxidiene treatment

Before the beginning of the experiment, herboxidiene (#25136; Cayman, Ann Arbor, MI, USA) was dissolved in dimethyl sulfoxide (DMSO) and the final concentrations were 10 mM and 100 µM. After the 1-h adsorption of viruses at 4 °C, the A549 cells were washed with PBS and maintained in serum-free DMEM supplemented with trypsin (0.2 μg/mL) and 0.2 µM herboxidiene at 37 °C for 36 h. The control group was treated with DMSO at a final concentration of 0.2%.

### In vivo* virus infection model*

C57BL/6 female mice (age: 7 weeks) were obtained from BioLASCO (Taiwan). Six mice were included in each group regardless their gender. Mice were intranasally infected with a viral dose of 1 × 10^3^ plaque-forming units (PFU) in 30 µL of serum-free DMEM, as described by Chen et al. [44]. After the challenge, changes in body weight and survival were monitored daily. In compliance with ethical standards and regulations for animal experiments, the end point “mortality” was considered to be reached at a weight loss of > 30% from the initial value.

### Splice site score analysis

The online tool MaxEntScan using the maximum entropy model was used to determine the splice site strength of the 5′ SS (http://hollywood.mit.edu/burgelab/maxent/Xmaxentscan_scoreseq.html) and 3′ SS (http://hollywood.mit.edu/burgelab/maxent/Xmaxentscan_scoreseq_acc.html) [45].

### Sequence information

M sequences were obtained from the Influenza Research Database (https://www.fludb.org/brc/home.spg?decorator=influenza) [46] and Global Initiative on Sharing Avian Influenza Data (GISAID) (https://www.gisaid.org) [47] accessed on November 7, 2019. A total of 30,897 sequences of human M IAVs were obtained, of which 18,500 of H1N1 and 12,397 of H3N2, as well as 19,709 and 5767 M sequences of avian and swine IAV isolates, respectively, were obtained and analyzed to determine the 55C/G and 740G/A frequencies and to design WebLogo [48] plots of the splicing sites. Subsequently, a total of 1120 sequences, including 503 H3N2 and 617 H1N1 strains, were randomly selected from different years and countries for further phylogenetic tree reconstruction. We also retrieved 52,339 H3N2 M sequences from the GISAID database (accessed on September 11, 2021) for the yearly distribution plot of 55T and 740A.

### Phylogenetic tree reconstruction

The Multiple Alignment using Fast Fourier Transform (MAFFT) tool (version 7.453) was used to align the sequences [49]. Bayesian evolutionary analysis by sampling trees (BEAST) (version 1.10.4) [50] using the Bayesian Markov chain Monte Carlo (MCMC) method was applied to infer the phylogenetic trees under the uncorrelated relaxed clock and General Time Reversible (GTR) plus gamma distribution substitution model. The MCMC chains were run for 107 generations in triplicate and sampled every 1000 steps. Runs were combined using LogCombiner software and all the effective sample size (ESS) values were greater than 200. Eventually, phylogenetic trees were annotated and visualized using the ggtree R package (version 3.2) [51].

### Statistical analysis

All data were expressed as mean ± standard deviation (SD). One-tailed paired *t*-test was used to compare the virus titer at every time point. Multiple comparisons between groups were performed using two-way analysis of variance. P < 0.05 was considered statistically significant. GraphPad Prism (version 8.2.0, GraphPad Software, La Jolla, CA, USA) was used for all statistical analyses.

## Results

### M *splicing of human IAVs varies in a subtype-specific manner*

**Fig. 1:**
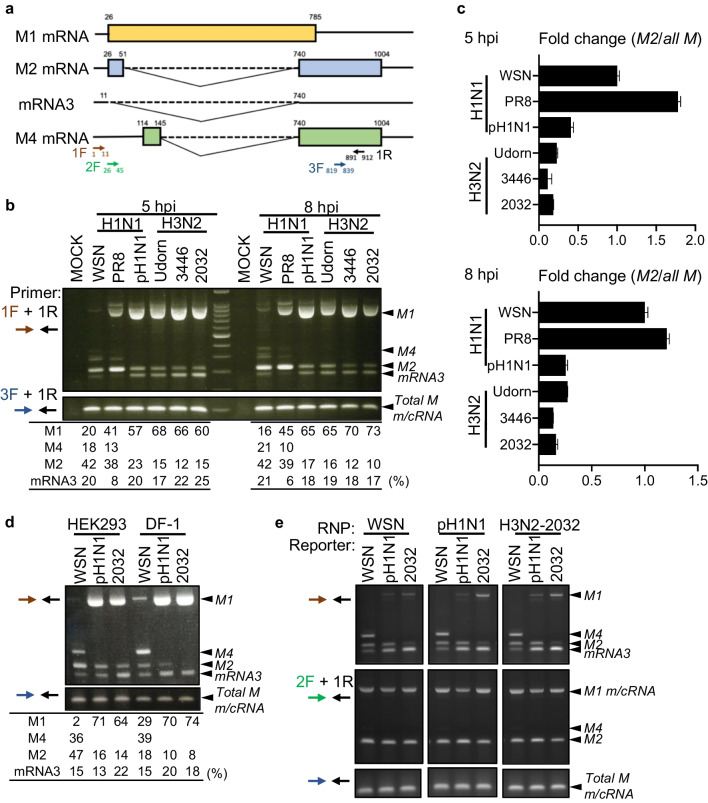
Preferential *M2* splicing in H1N1 over H3N2 influenza viruses. **a** Diagram of *M* segment splicing. The primers used are depicted (colored arrows). **b**, **c **Splicing pattern of *M* in HEK293 cells infected by six IAV strains (MOI = 1): A/WSN/33 (WSN), A/Puerto Rico/8/34 (PR8), A/TW/3773/2015 (pH1N1), A/Udorn/1972 (Udorn), A/TW/3446/2002 (3446), and A/TW/2032/2017 (2032). Total RNA was collected 5 and 8 h post-infection (hpi) and the splicing products were detected using RT-PCR (**b**) and qPCR (**c**). Fold change compared with WSN is expressed as the ratio of *M2* to all *M* mRNA. **d** HEK293 and DF-1 cells were infected with WSN, pH1N1, and 2032 (MOI = 1), and total RNA was collected at 5 hpi. **e** HEK293 cells were co-transfected with expressing plasmids encoding RNP (PB2, PB1, PA, and NP) and M reporter plasmids from different strains as indicated. After 48 h, total RNA was collected and analyzed. Densitometric analysis of each mRNA was quantified using ImageJ

We first examined the M splicing of human H1N1 and H3N2 viruses in human cells. Overall, six viruses—three H1N1 strains: A/WSN/33 (WSN), A/Puerto Rico/8/34 (PR8), and A/TW/3773/2015 (pH1N1); and three H3N2 strains: A/Udorn/1972 (Udorn), A/TW/3446/2002 (3446), and A/TW/2032/2017 (2032)—were compared. Total RNA was recovered 5- or 8-h post-infection and subjected to RT-PCR analyses using specific primers to detect the various spliced products of the M transcripts. Specifically, primer 1F_1–11_, in which extra GGGGG nucleotides were introduced at the 5′ end [52], was designed to distinguish all spliced isoforms; primer 2F_26–45_ amplified both *M2* and *M4*, and primer 3F_819–839_, targeting the common exon of all transcripts, served as transcription control (Fig. 1a). The splicing patterns of H1N1- and H3N2-infected cells differed regardless of the collection time. A preference for *M2* splicing was consistently observed in all H1N1 viruses, whereas fewer *M2* transcripts were detected in H3N2-infected cells (Fig. 1b). This *M2* enrichment in H1N1-infected cells was further validated by quantitative PCR (qPCR) using isoform-specific primers spanning each splice-site junction (Fig. 1b, c) [43]. We also examined the *M* segment splicing of human H1N1 and H3N2 viruses in chicken DF-1 cells. Consistently, only a few *M2* mRNAs were produced, irrespective of the virus subtype (Fig. 1d). Considering the importance of the intrinsic RNA polymerase in regulating *M* splicing [33, 35], we used the RNP reconstitution system to determine whether the splicing of M minigene reporters of the WSN (H1N1), pH1N1, and 2032 (H3N2) strains could be affected by swapping the polymerase complex with that of a different subtype. Overall, the splicing pattern of each reporter was similar to the infection outcome when the M reporter was co-transfected with the cognate RNP (Fig. 1b, e). Overexpression of the noncognate RNPs had no effect on strain-specific splicing (Fig. 1e); therefore, the different splicing efficiencies of H1N1 and H3N2 *M* segments may depend on the intrinsic *cis*-elements rather than the viral RNP.

### Identification of single nucleotide variations (SNVs) in the splice donor and acceptor sites of human IAV M2 segment

**Fig. 2:**
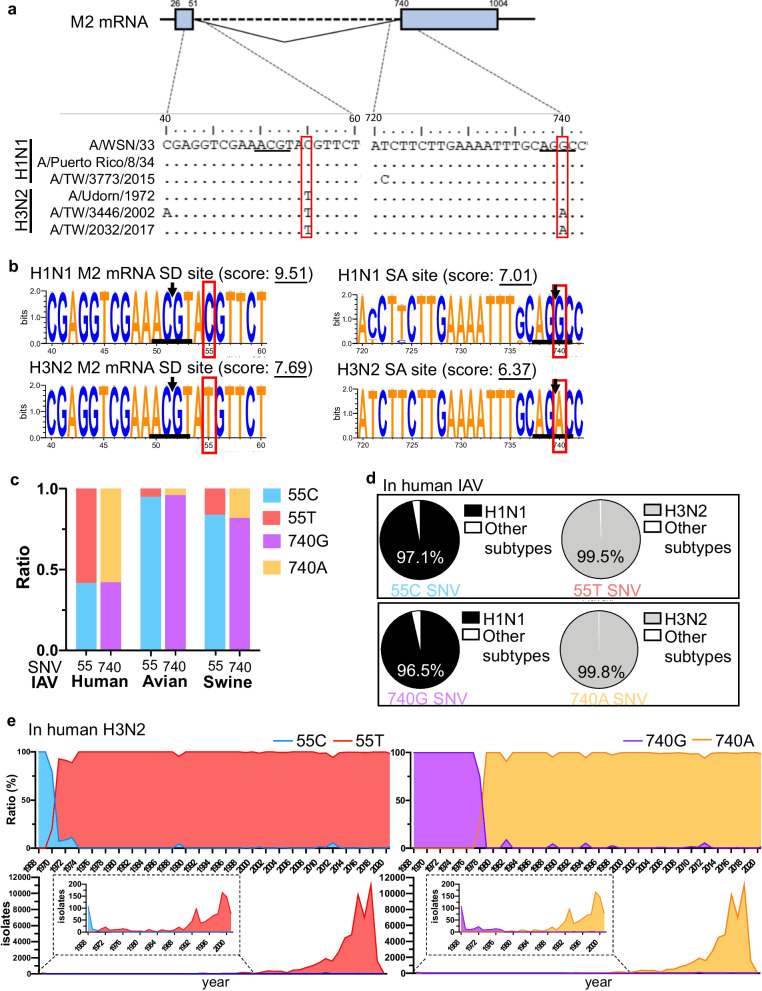
Single nucleotide variants contribute to different splice signals of *M* transcripts in human influenza A viruses (IAVs). **a** Alignment of the *M* segment of six IAV strains depicting different splice signals (underlined) in H1N1 and H3N2. The 55C to T and 740G to A variants are highlighted in black boxes. **b **WEBLOGO plots of the splice donor (SD; 5′ splice site; 5′ SS) and splice acceptor (SA; 3′ SS) of the *M* segment. The height of each base represents its frequency at a given position within the *M* sequences. The arrow indicates the position of the 5′ SS and 3′ SS in the SD and SA sites, respectively. Splice site strength was calculated by MaxEntScan (5′: 9 bp, − 3 to + 6; 3′: 23 bp, − 20 to + 3). **c** Frequency of sequences containing 55T/C and 740A/G in human, avian, and swine IAVs. The y axis of each panel represents the number of isolates containing the annotated nucleotide. **d** Percentage of the viral subtypes containing the annotated nucleotide. **e** Yearly distribution plot of 55T and 740A containing sequences of the human H3N2 subtype. The percentage is based on the *M* sequences of the H3N2 virus

**Fig. 3:**
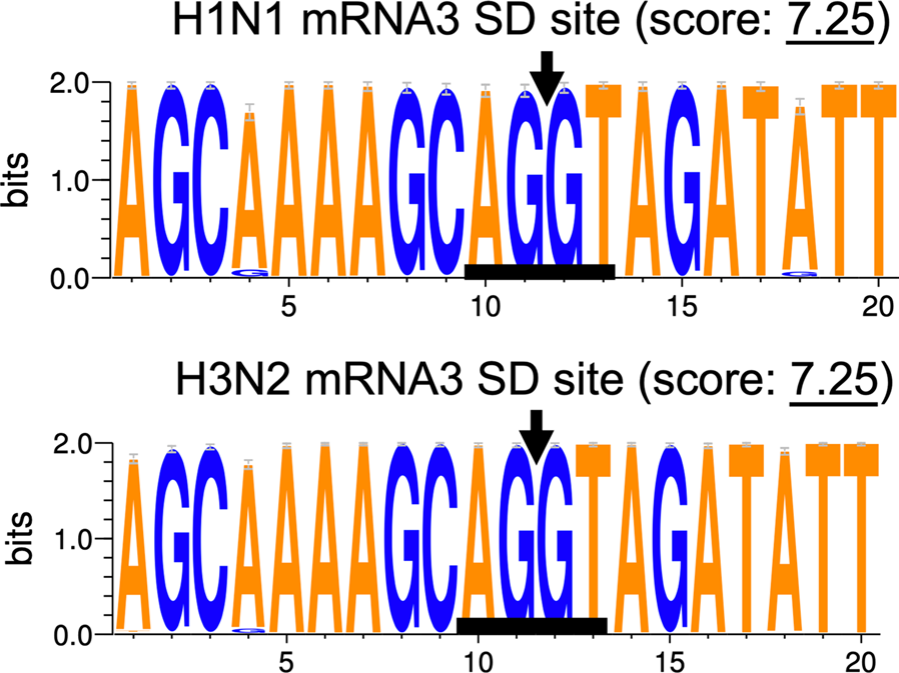
WEBLOGO plots of the *mRNA3* splice donor site of the *M* segment. The height of each base represents the frequency at a given position within the *M* sequences. The arrow indicates the position of the 5′ and 3′ splice site in the splice donor (SD) and splice acceptor (SA) sites, respectively. The splice site strength was calculated using MaxEntScan (5′ splice site: 9 bp, − 3 to + 6)

Considering the large difference in *M2* levels observed when the *M* splicing efficiency in the human H1N1 and H3N2 infected cells was compared (Fig. 1b), we analyzed the sequences spanning the *M2* splice donor (SD; the last 12 exonic plus first nine intronic nucleotides, − 12 to + 9) and splice acceptor (SA; the last 20 intronic plus first three exonic nucleotides, − 20 to + 3) sites. Pairwise alignment of the *M* cDNA of the six experimental strains showed that a single nucleotide substitution (C55T) close to the *M2* 5′ SS was present in all H3N2 strains, whereas another substitution (G740A) immediately downstream of the 3′ SS was identified in two of the H3N2 strains (Fig. 2a). Then, we performed an in silico analysis of the consensus sequence of the SD and SA sites using WebLogo [48]. A total of 31,110 M cDNA sequences downloaded from the online Influenza Research Database were analyzed, including 13,182 and 18,928 human H1N1 and H3N2 sequences, respectively. In contrast to the evolutionarily conserved SD of *mRNA3* (Fig. 3), the two substitutions identified in our experimental strains were found to be SNVs of the *M* sequences, in which the favored combinations in H1N1 and H3N2 were 55C–740G and 55T–740A, respectively. The 55C/T SNV strongly affected the splice site strength, with the splice site score being reduced from 9.51 to 7.69 by the T to C conversion (Fig. 2b). Considering the identical 5′ SS of *mRNA3* (with a splice site score of 7.25) carried by H1N1 and H3N2, the different *M2* splicing efficiencies might be a consequence of the altered splice site strength caused by 55C/T. Next, we analyzed the distribution of these SNVs in human, avian, and swine IAVs and found that they were only present in human IAVs. Both avian and swine IAVs were predominately associated with 55C and 740G and less than 18% of nonhuman influenza viruses had 55T–740A SNVs, suggesting that these SNVs may be human-specific (Fig. 2c). Characterizing the SNV-association of each subtype revealed that most human H1N1 (97.1%) and H3N2 (99.5%) genomes carried 55C and 55T, respectively, whereas 96.5% and 99.8% of human H1N1 and H3N2 genomes contained 740G and 740A, respectively (Fig. 2d). Examination of all publicly available sequences revealed that the H3N2 strains isolated earlier than 1970 also harbored 55C–740G; thus, the 55C to T and 740G to A switches approximately occurred in 1970 and 1980, respectively, and have been preserved since (Fig. 2e). As the first H3N2 virus appeared in the 1968 outbreak as a reassortment of the human H2N2 and avian H3 viruses (Fig. 2e), the SNVs of human H3N2 viruses may be acquired during evolution after reassortment.

### Adaptive evolution of the human H3N2 virus M segment from 1968 to 2019

**Fig. 4:**
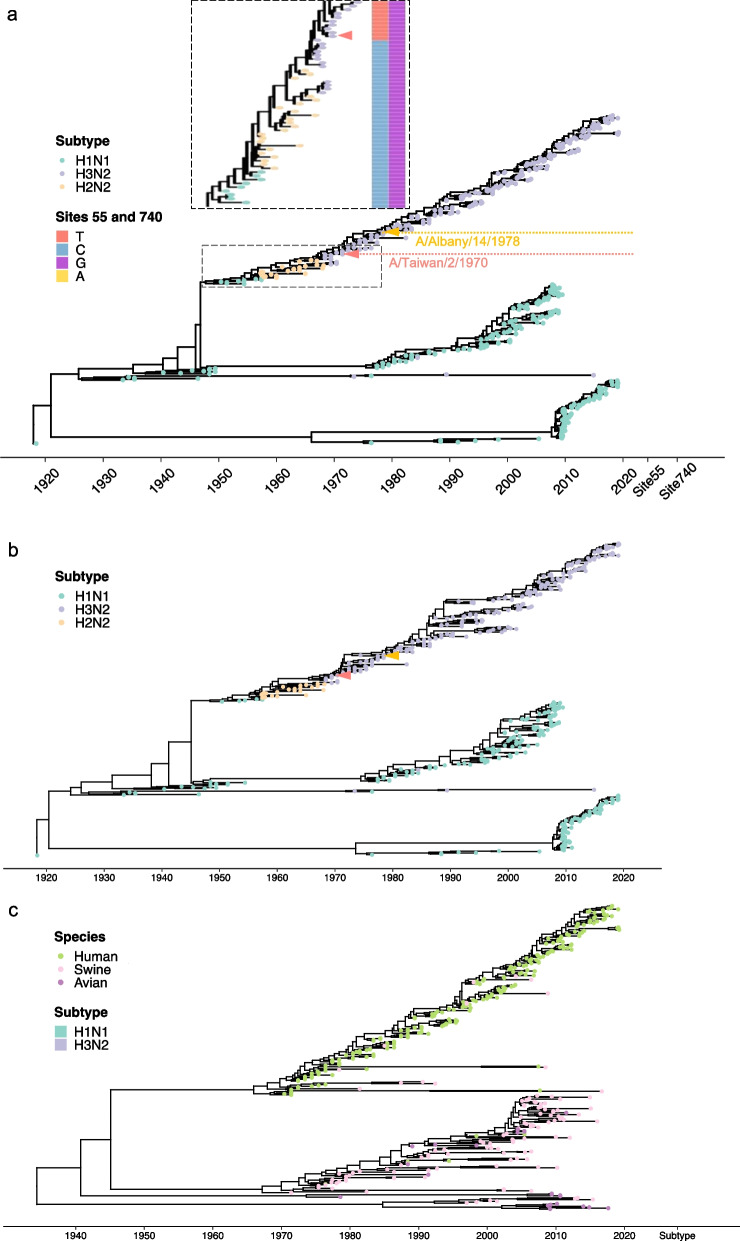
Phylogenetic analysis of influenza A virus (IAV) *M* segments. A phylogenetic tree was constructed based on (**a**) *M* segments of human IAV, (**b**) *M2* coding sequences of human IAV, and (**c**) *M* sequences of avian and swine IAV, and human H1N1 and H3N2. **a** The enlarged box shows the details regarding H3N2 emergence (purple circles) among sequences collected before 1970. The single nucleotide variations (SNVs) carried by each strain are denoted in the right-hand column. Orange and yellow arrow heads indicate the first incidence of the 55C to T and 740G to A switches, respectively. **c** The 55T-type isolates were selected from (**a**) (M/55T in H3N2 and H1N1 isolates). All sequences were aligned using MAFFT and analyzed using BEAST and TreeAnnotator [50]

**Fig. 5:**
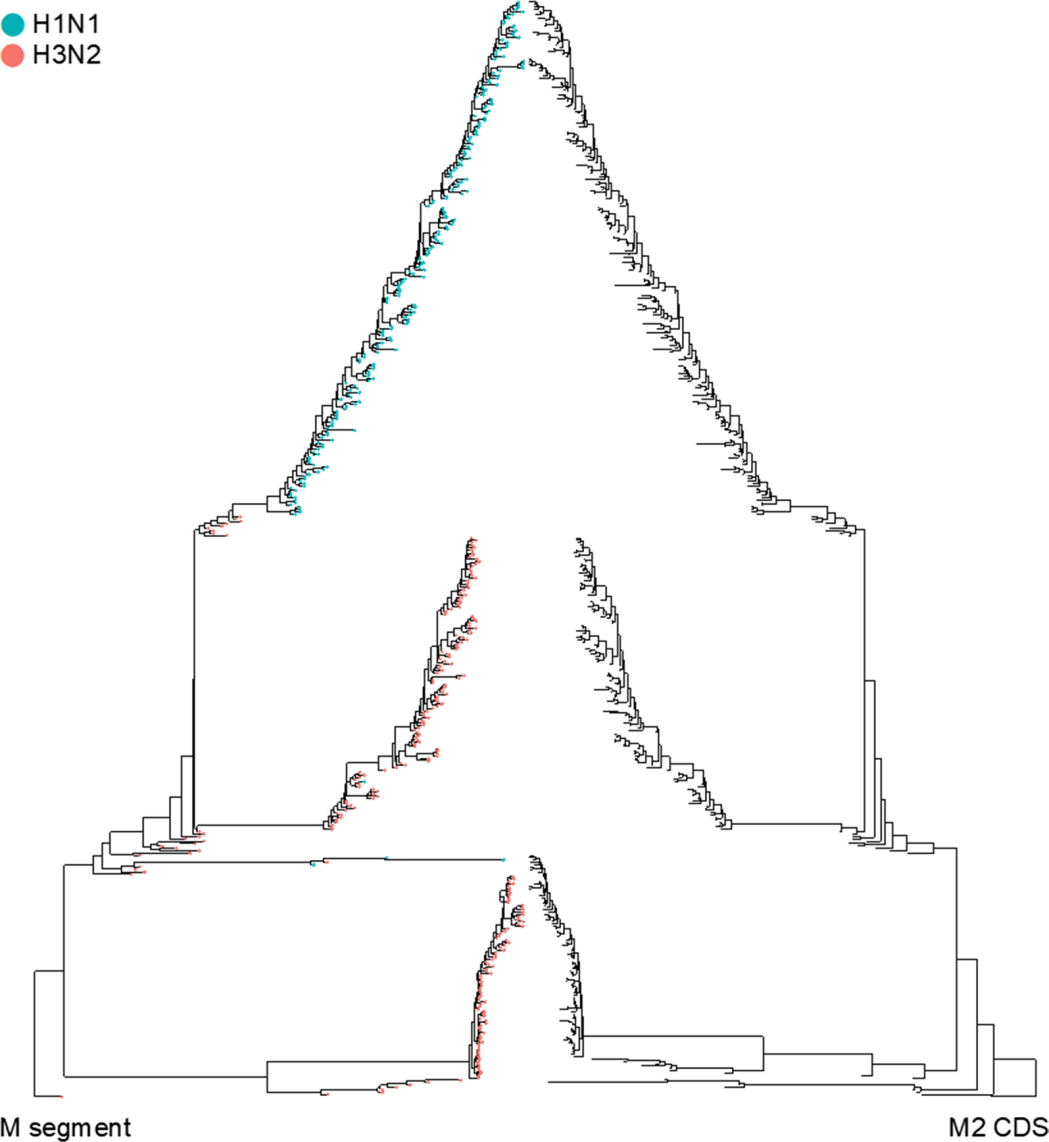
Concurrent evolution of the collinear *M1* and spliced *M2* isoform. Comparison between phylogenetic trees of the M segment and M2 coding sequence (CDS). Colors represent the subtypes and lines connect the same strains. The strains are correlated to Fig. 4a, b

To verify our hypothesis that SNVs may be a selection product to fine-tune the viral pathogenicity of the pandemic H3N2 virus to the following seasonal virus, we performed evolutionary analyses. We randomly selected *M* sequences of human H1N1, H2N2, and H3N2 viruses isolated from 1933 to 2020 and examined their phylogenetic relationships. Sequences of H2N2 were included because they are believed to have originated the H3N2 virus [53]. Two clusters were identified in the phylogenetic tree, which contained the *M* sequences that were dominant in H1N1 and H3N2 (Fig. 4a). The H3N2-dominant cluster showed an evolutionary path from H1N1 (blue circles) to H2N2 (yellow circles), followed by H3N2 (purple circles), as denoted by the ladder-like tree structure (Fig. 4a). Among the sequences collected before 1970 that harbored the 55C–740G SNVs, including ancient H1N1, all H2N2, and elder H3N2 sequences, the earliest C to T conversion identified (strain A/Taiwan/2/1970) was located within the H3N2 cluster. Moreover, the transition of 740A to G occurred in 1978 (strain A/Albany/14/1978), and almost all sequences (99.96%, Table 2) collected thereafter carried the 55T–740A SNVs (Fig. 4a). Hence, the H3N2-specific 55T–740A trait was not acquired from H2N2 reassortment (Fig. 4a). Notably, preservation of the 55T–740A trait did not change *M1* coding, that is, 55T/C and 740A/G SNVs are null and silent mutations, respectively. In contrast, the 55T-740A trait dramatically altered the yield of the *M2* isoform, which may be the selected product of evolutionary pressure. Therefore, we further examined the evolutionary relationship of M2 proteins among different human IAVs. Using an identical sequence pool for analyzing *M* sequences (Fig. 4a), the M2 coding sequences were extracted for evolutionary analysis. A highly similar tree structure of the M2 sequence was revealed, with most H3N2 M2 being grouped within an independent cluster (Fig. 4b). The concurrent evolution of the collinear *M1* and spliced *M2* isoform (Fig. 5) suggested that the functional impact of the 55T-740A SNVs may rely on the amount and/or function of M2. In addition to intra-species evolution, we examined inter-species evolution to determine whether H3N2 could acquire SNVs due to cross-species reassortment because a small portion of avian and swine IAVs also carry the 55T-type SNV (Fig. 2c; Table 2). Considering that most of the 740A-containing sequences were accompanied by the 55T SNV in human H3N2 (Table 2), we only traced the 55T variant in human, swine, and avian H1N1 and H3N2 (Fig. 4c). The 55T-type *M* segments could be separated into two clusters within the phylogenetic tree; one cluster contained all kinds of sequences, except for the human H3N2 sequences that were distributed in another cluster. The co-clustering of H1N1 sequences from different species indicated frequent reassortment events, whereas cross-species reassortment seldom occurred in human H3N2 (Fig. 4c). These results indicated that human H3N2 undertakes a unique evolutionary route depending on genetic drift rather than genetic shift. Overall, these findings suggest that the acquisition of 55T–740A mutations was spontaneous and preserved during the adaptive evolution of human H3N2. Next, we investigated the physiological roles of these SNVs in viral replication.

**Table 2:** Analysis of the composition of single nucleotide variations (SNVs) 55–740 (%)

	Human		Avian		Swine	
	H3N2	H1N1	H3N2	H1N1	H3N2	H1N1
55–740 SNV						
C-G	0.76	99.6	92.79	93.1	74.53	79.51
T-A	98.91	0.01	0	0	1.51	0.88
C-A	0.04	0	0.32	1.55	0	0
T-G	0.29	0.39	6.89	5.44	23.96	19.61
740A SNV						
T-A	99.96	100	0	0	100	100
C-A	0.04	0	100	100	0	0

A total 52,385 human H3N2, 13,178 human H1N1, 305 avian H3N2, 515 avian H1N1, 1916 swine H3N2 and 2162 swine H1N1 sequences were analyzed within sites 55–740. In the 55–740 SNV table, four different compositions were calculated. In the 740A SNV table, only sequences which had 740A SNV were calculated.

### Human M segment 55C/T variant regulates the expression of M2 isoform

**Fig. 6:**
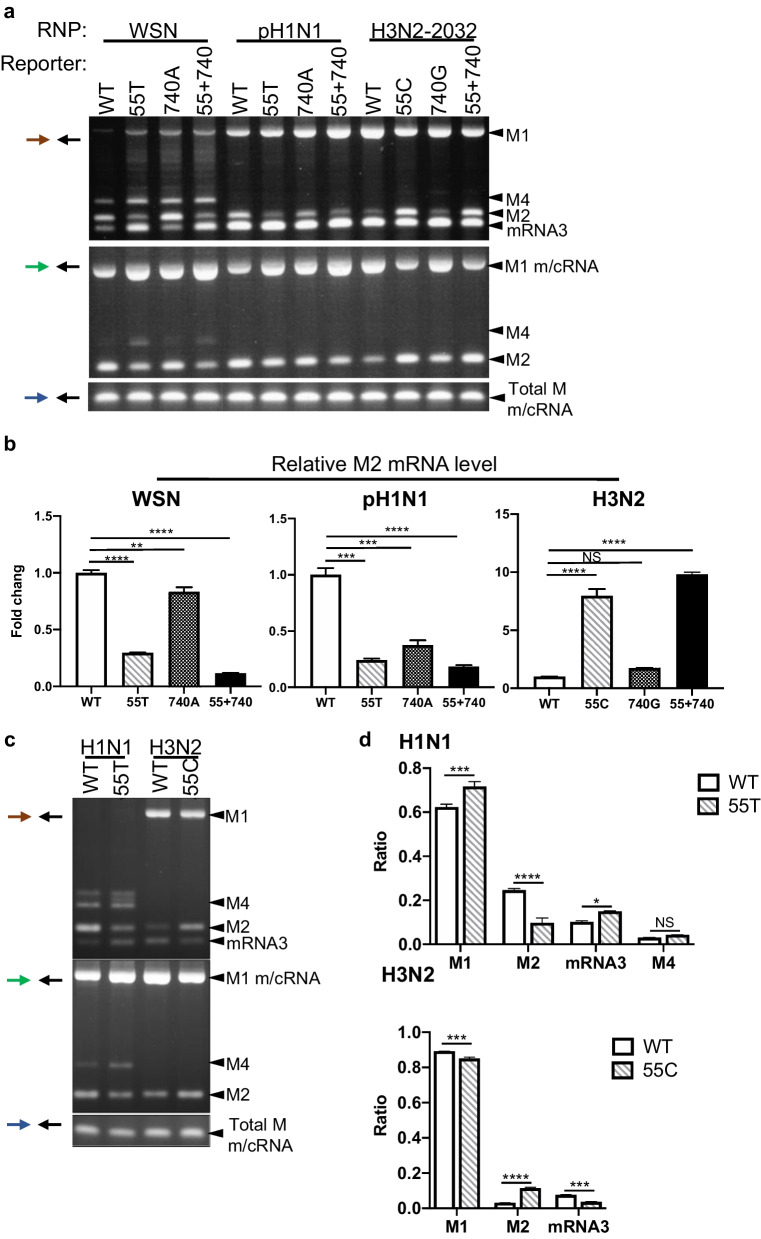
The 55 nucleotide is critical for the regulation of *M2* splicing. Splicing of *M* reporters bearing each single nucleotide variant (SNV) was assessed by RT-PCR (**a**) and qPCR (**b**) using specific primers (as shown in Fig. 1e). WT: wild-type M segment, 55T or 55C: M segment with 55 mutations, 740A or 740G: M segment with a 740 mutation, 55 + 740: M segment with double mutations. **c** *M* splicing pattern HEK293 cells infected by RG viruses (MOI = 1) for 5 h. WT: wild-type virus, 55T or 55C: an RG virus containing 55 mutations. **d** Relative expression of *M1*, *M2*, *mRNA3*, and *M4* determined using qPCR. Multiple comparisons between groups were performed using one-way analysis of variance. F values = 622.5 (WSN), 200.8 (pH1N1), and 411.9 (H3N2). Degrees of freedom (DFn, DFd) = (3,4). The data represent means ± standard deviations (error bars) of three independent biological replicates. NS, not significant; *P < 0.05, **P < 0.01, ***P < 0.001, and ****P < 0.0001

We first validated whether SNVs could affect the alternative splicing of *M* transcripts through RNP reconstitution assays using *M* reporter plasmids of H1N1 and H3N2 carrying the 55C/T and 740G/A variants. In the H1N1-based assays, introduction of 55T–740A (that is, the H3N2 trait) led to reduced *M2* expression, whereas the *mRNA3* and *M4* isoforms were increased. We attributed the aberrant splicing pattern to the single C55T mutation because the G740A mutation alone only moderately reduced the *M2* mRNA yield (Fig. 6a, b). When the H3N2 reporter was mutated to carry the H1N1 trait (that is, 55C–740G), we observed a more than tenfold increase in *M2* splicing efficiency in a T55C-dependent manner (Fig. 6a, b). These results demonstrate that 55C/T variation can modulate *M2* 5′ SS utilization and that 55C favors *M2* inclusion. In addition to the cell-based reporter assay, we determined the effects of the SNVs on *M* splicing in real infection by generating mutant viruses harboring the 55C/T variant through reverse genetics (RG). Regardless of the genotype, RG viruses bearing 55T were consistently characterized by a lower *M2* level (Fig. 6c). *M2* expression in the C55T mutant H1N1 virus was significantly reduced by approximately 50%. qPCR analysis revealed that collinear *M1*, *mRNA3*, and WSN-specific *M4* were increased in response to the decreased *M2* expression (Fig. 6d). Hence, aberrant *M2* splicing mediated by the 55C/T variant occurs through alternative 5′ SS utilization rather than a general defect in splicing processing. Given that *M* splicing efficiency has been proposed to regulate host restriction [22–24], we wondered whether the different splicing efficiency observed between H1N1 and H3N2 *M* affects viral replication, and whether the SNVs acquired by H3N2 could be beneficial for its adaptation in humans.

### C55T mutation attenuates the infectiveness of the WSN strain and reduces M2 protein levels

**Fig. 7:**
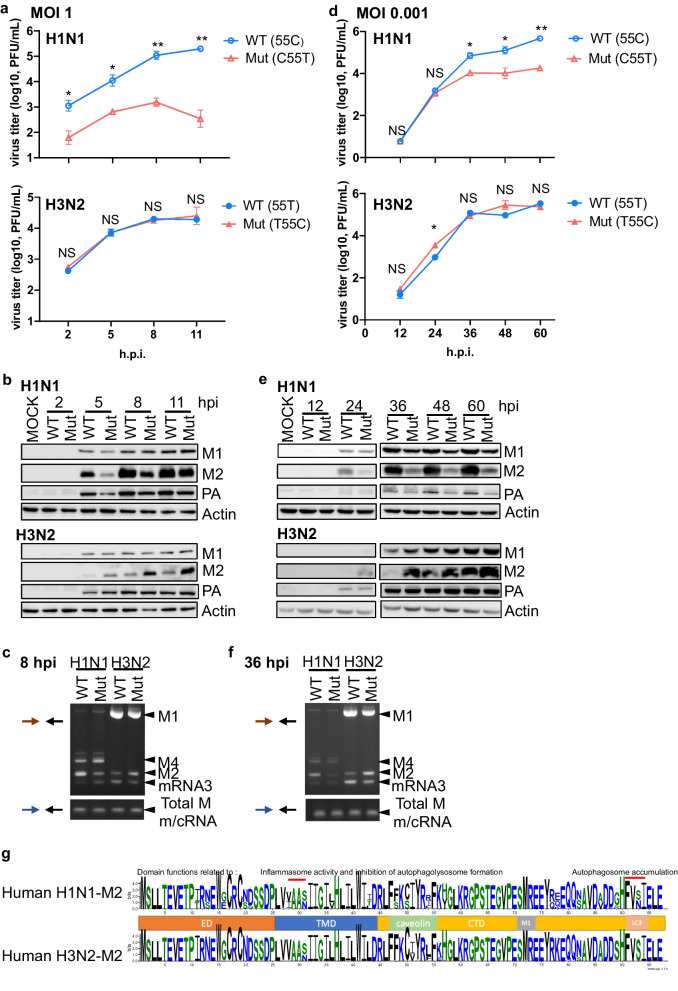
Reduced M2 protein due to 55T mutation leads to attenuated H1N1 virus in vitro. A549 cells were infected at a MOI of 1 or 0.001 with 55T- or 55C-type RG viruses. **a**, **d** The virus titer was analyzed by quantifying the number of plaques formed at the indicated time points (hpi, hours post-infection). **b**, **e** Protein expression was determined by western blotting using specific antibodies. Total RNA was collected after 5 (**c**) or 36 (**f**) h of infection. 1F and 1R primers were used to detect different *M* transcripts, and 3F and 1R primers were used to determine the expression of all *M* transcripts. Statistical analysis was performed using paired *t*-tests. **g** WEBLOGO plots of M2 amino acid sequences based on 242 human H1N1 and 222 human H3N2 isolates. The height of each amino acid represents the corresponding frequency. Protein domains of the M2 protein are depicted. The red line shows the domain region related to the above indicated functions. **a** H1N1 group, *t* = 5.001, df = 3 and H3N2 group, *t* = 0.4146, df = 3. **d** H1N1 group, *t* = 2.501, df = 4 and H3N2 group, *t* = 1.361, df = 4. (*t* = *t*-value, df = degrees of freedom). The data represent the mean ± standard deviation (error bars) of three independent biological replicates. NS, not significant; *P < 0.05; and **P < 0.01

As RG viruses bearing different SNVs were associated with altered *M2* expression, we specifically investigated the impact of variable *M* splicing on viral replication. H1N1 (WSN) RG viruses prepared from HEK293 cells were used to infect A549 cells at a MOI of 1 or 0.001 (Fig. 7a, d). When viruses harboring the C55T mutation were examined, approximately one log_10_ lag in viral growth was observed regardless of the MOI, and the above described SNV-induced splicing pattern was conserved in A549 cells (Fig. 7c, f). As expected, the levels of H1N1 M2, but not H1N1 M1, were consistently reduced during the infection due to impaired splicing (Fig. 7b, e), suggesting that reduced M2 levels may attenuate the infectiveness of H1N1 viruses. Impaired replication was also confirmed by the lower protein levels of PA. However, this M2 dependence was not observed in H3N2 (2032) RG viruses. Although the T55C mutation in H3N2 reciprocally enhanced the *M2* splicing efficiency, as well as the M2 protein level during infection (Fig. 7b, c, e, f), the H3N2 RG viruses grew to a similar extent regardless of which SNV was introduced (Fig. 7a, d). Thus, in contrast to the H1N1 viruses, disturbance of the M2 levels apparently has a minor effect on H3N2 replication. However, the importance of M2 on H3N2 cannot be assessed by this discrepancy.

### Inhibition of human IAV replication by the splicing inhibitor herboxidiene

**Fig. 8:**
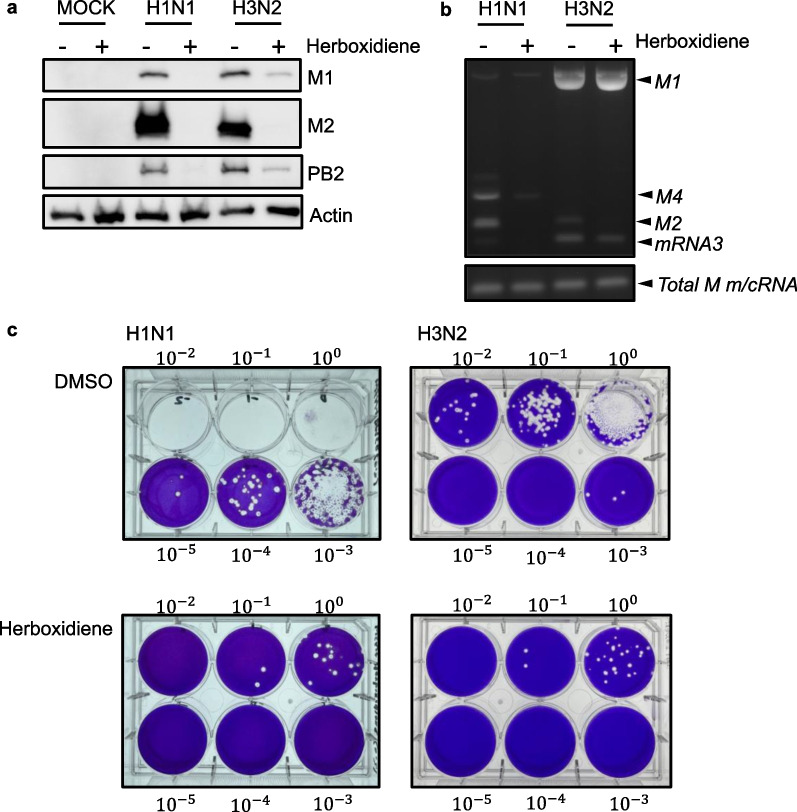
Herboxidiene efficiently inhibits the replication of influenza A virus (IAV). A549 cells were infected at a MOI of 0.001 with H1N1 (A/WSN/33) or H3N2 (A/2032/2017) virus after 36 h of infection. **a** Protein expression was determined by western blotting using specific antibodies. **b** Total RNA was collected and identified using RT-PCR, 1F and 1R primers were used to detect different *M* transcripts, and 3F and 1R primers were used to determine the expression of all *M* transcripts. **c** Plaque formation was visualized through crystal violet staining

To confirm the importance of *M2* splicing in the replication of both H1N1 and H3N2, we treated infected cells with herboxidiene, a potential antitumor drug that inhibits splicing. Both the M1 mRNA splicing and M2 protein expression of IAV viruses were downregulated after herboxidiene treatment, regardless of the subtype (Fig. 8a, b). Moreover, viral protein PB2 was downregulated in H3N2 and even disappeared in H1N1 infected cells, suggesting the inhibited replication of both viruses. The virus titer of both viruses decreased dramatically (Fig. 8c), and it declined from ${10}^{5}$ to ${10}^{1}$ pfu/mL in H1N1, whereas a 2 log decrease was observed in H3N2. Although the replication of H1N1 and H3N2 was affected to a different extent after herboxidiene treatment (Fig. 8b, c), M2 proteins were critical for the growth of both H1N1 and H3N2 viruses. These results indicated that splicing inhibitors may be regarded as potential therapeutic agents against influenza virus infection.

### Compatibility of WSN replication with the optimal protein level of H3N2 M2

There might be several underlying mechanisms causing the discrepancy in M2-dependence. First, the functionality of H1N1 and H3N2 M2 may differ, and a low level of H3N2 M2 may function as well as a high level of H1N1 M2. Second, aberrantly spliced products accompanying the weakened M2 5′ SS may compensate for M2 function. Third, other co-variants yet to be identified in H3N2 may contribute to the tolerance of reduced M2. However, 55C/T SNV is a synonymous substitution for the coding of non-M2 isoforms, whereas variant amino acid residues in M2 of H1N1 and H3N2 were identified as important domains for NLRP3 inflammasome activation and the subversion of autophagy machinery [29] (Fig. 7g). Therefore, we pursued the hypothesis that the protein activity of H1N1 and H3N2 M2 might differ, leading to the difference in M2-dependence.

**Fig. 9:**
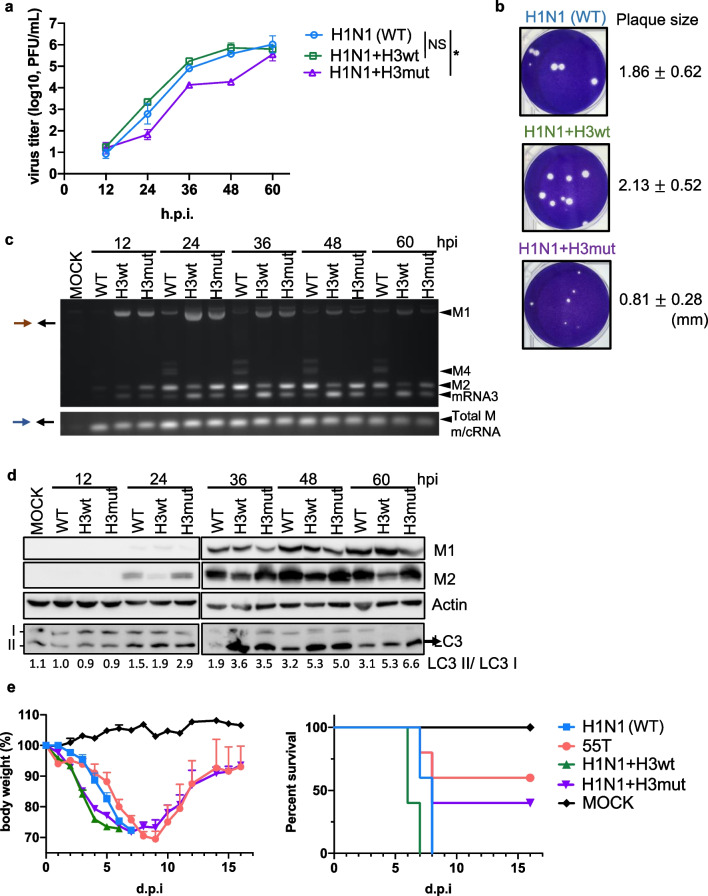
Compatibility of H1N1 virus replication with the optimal level of H3N2 M2. **a** A549 cells were infected with the H1N1 WSN strain (WT) or with chimeric H1N1 RG viruses incorporating either WT 55T (H1N1 + H3wt) or mutant 55C (H1N1 + H3mut) H3N2 *M* segments, at a MOI of 0.001. The virus titer was determined at 12, 24, 36, 48, and 60 h post-infection (hpi) through plaque assay. The data represent means ± standard deviations (error bars) of three independent biological replicates. NS, not significant; *P < 0.05. Statistical analysis was performed using paired *t*-tests. WSN verses WSN + H3mut groups, *t* = 2.435, df = 4 and WSN verses WSN + H3wt groups, *t* = 2.002, df = 4. (*t* = *t*-value, df = degrees of freedom). **b** Plaque formation was visualized through crystal violet staining, and plaque size was determined using ImageJ. **c** Total RNA was collected at the indicated time points. 1F and 1R primers were used to detect different *M* transcripts, and 3F and 1R primers were used to determine the expression of all *M* transcripts. **d** Total protein extracts were analyzed by western blotting using specific primers. **e** C57BL/6 mice were intranasally infected with WT, mutant H1N1 (55T), H1N1 + H3wt, and H1N1 + H3mut, or administrated control vehicle (mock). Survival rate and body weight were assessed daily. All data were normalized to the initial weight of each mouse. Data are expressed as mean ± standard error of the mean (n = 5 mice per group) and three independent biological replicates

To confirm this, we examined the compatibility between M2 proteins of H1N1 and H3N2. We generated chimeric H1N1 RG viruses by incorporating either the wild-type (WT) 55T (H1N1 + H3wt) or mutant 55C (H1N1 + H3mut) H3N2 *M* segment instead of the H1N1 *M* segment. Infection with these chimeric RG viruses was performed in A549 cells at a MOI of 0.001, and these cells were compared with those infected with WT H1N1 RG viruses. Although a much lower M2 protein level was detected in cells infected by H1N1 + H3wt than in cells infected by H1N1, similar virus replication rates were observed (Fig. 9a, c, d), indicating that the lower levels of preserved H3N2 *M* segment were compatible with H1N1 replication, at least in the WSN strain. In contrast, replication of the H1N1 + H3mut virus, which was characterized by the elevated levels of M2, was significantly impaired (Fig. 9a, c, d), suggesting that the T55C mutation in H3N2 *M* was not beneficial for H1N1 replication. Notably, the plaque size of the chimeric H1N1 + H3mut was considerably smaller than that of the H1N1 wild-type and H1N1 + H3wt chimeric viruses (Fig. 9b). To validate the splicing pattern, total RNA of the infected cells was collected at various time points and analyzed using RT-PCR. *M2* transcripts were the dominant isoform in cells infected with wild-type H1N1 viruses throughout the infection (Fig. 9c). In contrast, infection of the chimeric H1N1 + H3wt virus was characterized by decreased M2 splicing (Fig. 9c). The T55C mutation in the H3N2 *M* segment considerably switched the splicing from *mRNA3*-dominant to *M2*-dominant (Fig. 9a, lane H1N1 + H3mut**)**. Therefore, the regulation of *M* splicing by SNVs in authentic H3N2 viruses can be recapitulated in heterogeneous chimeric H1N1 RG viruses (Figs. 7c, f, 9c). The preserved *M* segment splicing pattern of H3N2 in the presence of H1N1 viral proteins not only emphasizes the importance of the 55C/T SNV, but also excludes the possibility that altered *M* splicing can be mediated by other viral proteins, such as NS1. Consistent with the changes in the mRNA level, fewer M2 proteins were detected in H1N1 + H3wt virus-infected cells, whereas M2 was elevated along with a concomitant decrease in the M1 in cells infected with the H1N1 + H3mut virus (Fig. 9d). We observed that low expression of H3N2 M2 efficiently activated LC3 cleavage as compared to that of H1N1 M2 (Fig. 9d). The efficient LC3 cleavage by M2 might partly explain the deleterious effect of excess H3N2 M2 in the non-cognate H1N1 virus.

### Optimized M2 expression in a subtype-specific manner determines IAV pathogenicity

To explore the physiological role of the various levels of M2 in IAV pathogenicity, we challenged mice with the H1N1 WSN strain (WT), mutant H1N1 (55T), and chimeric viruses including H1N1 + H3wt and H1N1 + H3mut. Less body weight loss was observed in mice challenged with the H1N1-55T mutant virus. Moreover, H1N1-55T-infected mice showed a significantly better survival rate than WT H1N1-infected mice (60% vs. 0%) (Fig. 9e). Consistent with the attenuated replication of the mutant H1N1 virus in A549 cells (Fig. 7a, d), the pathogenicity of the H1N1-55T virus was also attenuated. All mice challenged with the chimeric H1N1 + H3wt virus died with a similar disease progression to those challenged with the WT H1N1 virus (Fig. 9e), whereas the survival rate of the H1N1 + H3mut infected group dramatically improved from 0 to 40%. These results clearly demonstrated that the functionality of H3N2 M2 was different from that of H1N1 M2, thus contributing to different pathogenicity. In addition, the H3N2-specific 55T variant may have evolved to maintain the optimal level of M2 with the appropriate activity required for virus replication. In addition, human H1N1 viruses, and probably IAVs of other species, may require more M2 proteins for virus replication. Thus, optimization of M2 proteins with respect to their functionality may play a pivotal role in the replication of IAVs.

## Discussion

**Fig. 10:**
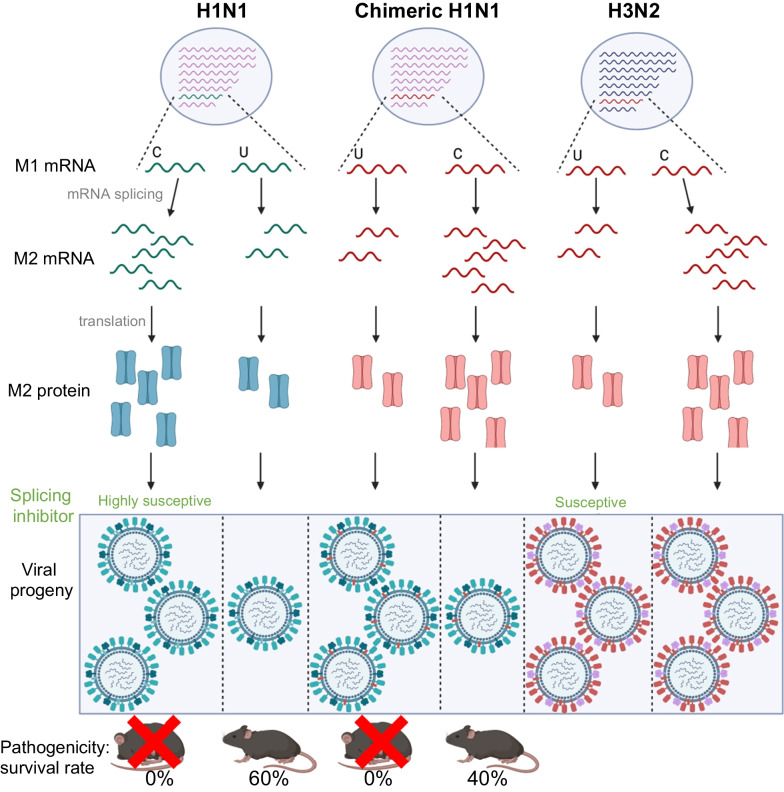
Hypothesis model of this study. Evolutionary selection has favored the 55C single nucleotide variant (SNV) trait of the *M* segment of the H1N1 virus, which promotes the splicing efficiency of *M2* and consequent upregulation of M2 expression. While the 55C site changes to 55T, the splicing of *M2* is inhibited, thereby leading to low M2 expression and impaired H1N1 virus replication. However, the H3N2-specific 55T variant results in low M2 protein expression. Even though the T55C trait can help express more H3N2 M2, it has no effect on the replication of the virus. Using chimeric viruses, low H3N2 M2 levels are enough to rescue the replication rate similar to that of wild-type H1N1 virus. However, excessive H3N2 M2 seems to be harmful for H1N1 virus. Noteworthily, SNVs can also show impact on the pathogenicity of influenza A viruses (IAVs) in vivo. 55T-typed H1N1 viruses showed the highest survival rate among all H1N1 viruses

We identified a *cis*-acting 55C/T SNV that is unique to human IAV and modulates the *M* segment splicing. The human H3N2 virus is characterized by a lower splicing efficiency of *M2* under both infection and RNP reconstitution conditions. By generating different subtypes of RG viruses harboring distinct SNVs, we demonstrated that a mutation at the 55-nucleotide position impairs the replication of H1N1 but not of the H3N2 virus. Moreover, we discovered that the low level of H3N2 M2 fulfills the functional requirement of H1N1 M2 in the chimeric RG viruses, whereas elevated H3N2 M2 protein levels induced by the T55C mutation have deleterious effects on H1N1 replication. Mice challenged with the C55T mutant H1N1 virus showed an improved survival rate and reduced weight loss as compared with those infected with wild-type viruses. Notably, the pathogenicity of chimeric H1N1 viruses remained unchanged if the wild-type H3N2 *M* segment was present; however, milder symptoms developed when mice were infected with chimeric viruses carrying the mutant H3N2 *M* segment. The discrepancy in the M2-dependence is unique to human IAVs, which are derived from an adaptive evolution route undertaken by H3N2 *M* (Fig. 10).

Viruses evolve by accumulating mutations that support the adaption to the human host, such as stronger receptor binding features and better RNP activity [54, 55]. The high mutational rate of IAVs also compromises the host antiviral activity. Therefore, continuous surveillance of seasonal IAV infections is necessary. However, few studies have compared the pathogenicity of human H1N1 and H3N2, and the specific traits and mutations contributing to their differences in transmission and infectivity remain poorly characterized. The hypothesis of a strain-specific trait that could alter virulence was first suggested by Yamayoshi et al. [4], who found a spliced *PB2-S1* that only existed in cells infected by pre-2009 H1N1, but no by H3N2 or current H1N1. Our findings showing the variable splicing efficiency of the H1N1 and H3N2 *M* segments serve as another example. The H3N2 virus carries a weak 5′ SS for *M2*, leading to low M2 protein expression during infection (Figs. 2b, 7b, e). We discovered that the differences in *M* splicing contribute to infectiveness and pathogenicity variations (Figs. 7, 8, 9). We also showed that H3N2 underwent strain-specific evolution (Fig. 4); however, how the virus can tolerate low *M* splicing remains to be explored. The concurrent evolution of the collinear *M1* and spliced *M2* sequences in H3N2 raises the possibility that M2 protein, rather than M1, may be subjected to selection pressure. Therefore, by decreasing the M2 levels, the 55T trait is preserved in H3N2 to support the optimized activity required for viral replication.

**Fig. 11:**
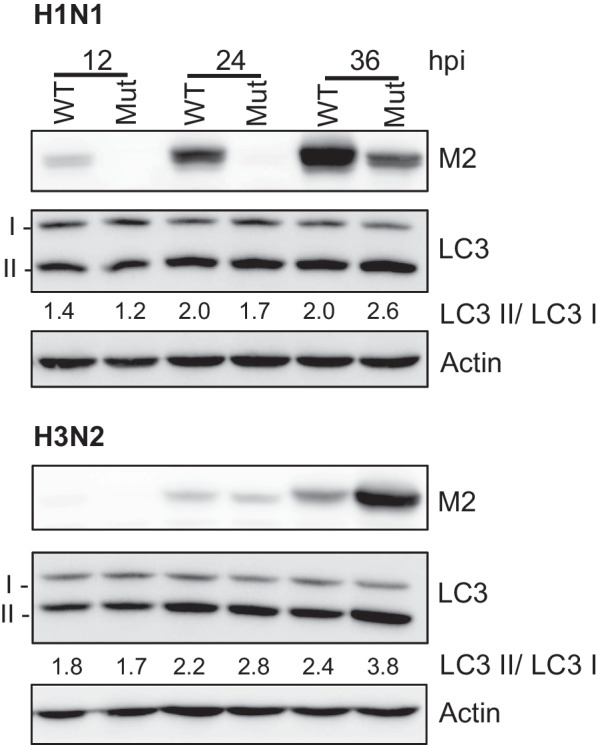
Low expression of H3N2 M2 can efficiently activate LC3 cleavage. Western blot showing expression of viral proteins using specific antibodies. A549 cells were infected by H1N1 or H3N2 at MOI 0.001, and cell lysates were collected at 12, 24, and 36 h post-infection. Top panel: H1N1 wild type (WT) (M/55C) and mutant (Mut) (M/C55T). Bottom panel: H3N2 WT (M/55T) and Mut (M/T55C). Densitometric analysis was performed using ImageJ software

Ectopically-expressed M2 protein alone can activate inflammasomes and subvert the autophagy pathway. M2 binds to LC3 and inhibits the fusion of autophagosomes with lysosomes to counteract clearance of the virus; thus, cleaved LC3 gets accumulated (Fig. 11) [30]. However, the role of autophagy in viral replication remains controversial. Although the proviral role of autophagy in IAV replication has been proposed [56–58], it has been shown that inhibition of autophagy does not affect IAV infection [28, 59], and that the interaction between M2 and autophagy components may support viral replication [57]. Therefore, the splicing regulation of the *M* segment has been proposed as a host-restriction mechanism [22–24]. Considering the different virus subtypes used in these studies, the importance of subtype-specific factors is further highlighted. Our findings indicate that, in addition to the protein level, the functionality of different subtypes of M2 should be considered. We provided evidence that low expression of H3N2 M2 can efficiently activate LC3 cleavage (Fig. 9d and 11). Therefore, the compromised replication of the chimeric H1N1 virus by the overproduction of H3N2 M2 may be attributed to imbalanced regulation. Nevertheless, whether the discrepancy in the M2-dependence observed in human IAVs is caused by the differential capacity to subvert autophagy or other mechanisms yet to be identified is worth pursuing in the future.

The rapid emergence of drug-resistant viruses complicates the application of current anti-influenza drugs [60]. Therefore, there is an urgent need to develop novel strategies to combat influenza. In our study, diminishing the *M2* splicing efficiency of H1N1 impaired both the replication and pathogenicity of the virus (Figs. 7a, d, 9e). TG003, an inhibitor of the splicing regulator CLK1, is known to inhibit influenza virus infection by blocking *M* splicing [34, 37, 39]. We found that the M2 dependence of the H3N2 virus was less than that of H1N1, which raises concerns about the splicing dependence of human H3N2. However, treatment with herboxidiene inhibited H1N1 and H3N2 replication possibility through the downregulation of M2 production. It has been shown that influenza viruses can tolerate NS1-deficiency and their growth was severely decreased by approximately 3 logs [61]. In this study, we found an approximate decline of 4 logs in the herboxidiene-treated group, indicating the H1N1 virus is highly dependent on M2 production. Splicing modulation is one of the current cancer treatment strategies. Herboxidiene, which is a natural product isolated from *Streptomyces* spp., showed significant antitumor activity in mice by targeting splicing factor SF3b1 [62]. An optimized molecule H3B-8800 (RVT-2001) is already in clinical trial phase (Trial Identifier: NCT02841540) [63], and its potential in antivirus treatment remains to be examined. In addition to using a splicing inhibitor, the targeting of M2 can be another strategy against influenza. The low M2 demand of H3N2 can be partly explained by the different protein functionalities. Although the M42 protein may compensate for the ion channel activity of M2 in the WSN strain [10], no evidence supports the existence of a M4 isoform in H3N2 and other viruses. [11]. Moreover, membrane-associated RING-CH proteins were reported to inhibit IAV replication through M2 degradation [64]. Overall, the importance of M2 cannot be ignored and targeting the *M* segment through splicing should be considered as a promising antiviral strategy.

In contrast to the well-preserved 55T SNV in human H3N2 since 1970, SNV was seldom detected in other IAVs and occurred sporadically and diminished soon after (Fig. 4). The different compatibility of the chimeric H1N1 RG viruses to high or low levels of H3N2 M2, which was not observed in H3N2 RG viruses, suggests that H3N2 may have evolved to tolerate the potent M2 protein. In addition, the human H3N2 virus seems to be accessible to the H1N1 *M* segment. Several H3N2 viruses harbor H1N1-origin *M* segments, as shown in Fig. 4c. Co-circulation of human H1N1 and H3N2 viruses occurred in 2010 [65], and a reassortant H3N2 variant virus carrying a pdmH1N1 *M* segment was initially isolated from pigs in 2010, and then caused an outbreak in the United States [21]. No H1N1 virus carries the *M* segment originating from H3N2, even though the reassortment of H1N1 and H3N2 can occur in nature. The disfavor of the H3N2 *M* segment in H1N1 is consistent with the laboratory finding that the H1N1 *M* was preferred in experimentally-forced reassortment of the H1N1 and H3N2 viruses [20]. However, whether the evolutionarily-conserved 55T trait in human H3N2 viruses is accompanied by any covariant remains unclear.

Finally, the roles of *trans*-factors in *M* segment splicing and host restriction should be considered. We showed that C55T conversion can dramatically affect *M* splicing in the RNP reconstitution system, irrespective of the virus subtype (Fig. 1e). In addition, the C55T mutation significantly inhibited the growth of WSN viruses. Therefore, it seems likely that *cis*-acting SNVs may bypass viral *trans*-factors. However, we cannot rule out the possibility that H1N1 and H3N2 may acquire different adaptations and strategies to overcome the host restriction factors that regulate the splicing of the *M* segment by recognizing the 55C/T trait.

## Conclusions

We discovered a 55T SNV in the human H3N2 virus that decreases the splicing efficiency of *M2*, resulting in reduced M2 expression. Compared with H3N2, H1N1 is more dependent on M2 yield during replication. However, the functionality of the M2 protein of both viruses may differ, as overexpression of H3N2 M2 protein inhibits the replication of H1N1 chimeric viruses. The adaptive evolution of human H3N2 illustrates that preservation of the 55T SNV confers a survival advantage to the virus. The difference between human H1N1 and H3N2 revealed in this study not only provides insights into virus adaptation processes in humans, but also highlights splicing regulation as a potential antiviral target.

## Data Availability

The datasets used and/or analyzed during the current study are available from the corresponding author on reasonable request.

## Abbreviations

5′ SS: 5′ Splice site
BEAST: Bayesian evolutionary analysis by sampling trees
MCMC: Bayesian Markov chain Monte Carlo
CLK1: Cdc-like kinase 1
DMSO: Dimethyl sulfoxide
DMEM: Dulbecco’s Modified Eagle Medium
ESS: Effective sample size
FBS: Fetal bovine serum
GTR: General time reversible
GISAID: Global initiative on sharing avian influenza data
HA: Hemagglutinin
hnRNP K: Heterogeneous nuclear ribonucleoprotein K
HEK293: Human embryonic kidney 293
IAVs: Influenza A viruses
NS1-BP: Influenza virus NS1-binding protein
MDCK: Madin–Darby canine kidney
MEM: Minimal essential medium
MAFFT: Multiple alignment using Fast Fourier Transform
Mut: Mutant
NA: Neuraminidase
NS1: Non-structural protein 1
NEP: Nuclear export protein
NP: Nucleoprotein
pdmH1N1: Pandemic H1N1
PBS: Phosphate-buffered saline
PFU: Plaque-forming units
PA: Polymerase acidic
PB1: Polymerase basic 1
qPCR: Quantitative PCR
RG: Reverse genetics
RT-PCR: Reverse transcriptase-polymerase chain reaction
RNP: Ribonucleoprotein
SRSF1: Serine/arginine-rich splicing factor 1
SNVs: Single nucleotide variations
SA: Splice acceptor
SD: Splice donor
SD: Standard deviation
TRA2A: Transformer-2 protein homolog alpha
vRNA: Viral RNA
WT: Wild-type
